# An overview of the gene regulatory network controlling trichome development in the model plant, *Arabidopsis*

**DOI:** 10.3389/fpls.2014.00259

**Published:** 2014-06-05

**Authors:** Sitakanta Pattanaik, Barunava Patra, Sanjay Kumar Singh, Ling Yuan

**Affiliations:** Department of Plant and Soil Sciences, Kentucky Tobacco Research and Development Center, University of KentuckyLexington, KY, USA

**Keywords:** gene regulation, microRNA, ubiquitin/26S proteasome, trichome

## Abstract

Trichomes are specialized epidermal cells located on aerial parts of plants and are associated with a wide array of biological processes. Trichomes protect plants from adverse conditions including UV light and herbivore attack and are also an important source of a number of phytochemicals. The simple unicellular trichomes of *Arabidopsis* serve as an excellent model to study molecular mechanism of cell differentiation and pattern formation in plants. The emerging picture suggests that the developmental process is controlled by a transcriptional network involving three major groups of transcription factors (TFs): the R2R3 MYB, basic helix-loop-helix (bHLH), and WD40 repeat (WDR) protein. These regulatory proteins form a trimeric activator complex that positively regulates trichome development. The single repeat R3 MYBs act as negative regulators of trichome development. They compete with the R2R3 MYBs to bind the bHLH factor and form a repressor complex. In addition to activator–repressor mechanism, a depletion mechanism may operate in parallel during trichome development. In this mechanism, the bHLH factor traps the WDR protein which results in depletion of WDR protein in neighboring cells. Consequently, the cells with high levels of bHLH and WDR proteins are developed into trichomes. A group of C2H2 zinc finger TFs has also been implicated in trichome development. Phytohormones, including gibberellins and jasmonic acid, play significant roles in this developmental process. Recently, microRNAs have been shown to be involved in trichome development. Furthermore, it has been demonstrated that the activities of the key regulatory proteins involved in trichome development are controlled by the 26S/ubiquitin proteasome system (UPS), highlighting the complexity of the regulatory network controlling this developmental process. To complement several excellent recent relevant reviews, this review focuses on the transcriptional network and hormonal interplay controlling trichome development in *Arabidopsis*.

## INTRODUCTION

Trichomes are epidermal protuberances that, depending on species, are located on the aerial parts of plants such as the leaves, stems, petioles, petals, and seed coat. They are generally classified into two types: simple, or non-glandular, and glandular secreting trichomes (GSTs; Wagner et al., 2004). Trichomes play an important role in plant growth and development by protecting them from UV light, insect predation, and excess transpiration. The phytochemicals secreted by GSTs provide protection against pathogens and pests, and also attract pollinators (Wagner, 1991; Wagner et al., 2004; Schilmiller et al., 2008). GSTs can also be considered “chemical factories” as they synthesize and secrete many economically important compounds (Schilmiller et al., 2008). The unicellular non-glandular trichomes of *Arabidopsis* serve as an excellent experimental system to study molecular mechanism of cell differentiation and pattern formation in plants.

The production and distribution of trichomes is spatially and temporally controlled. During the early vegetative phase, trichomes are present only on the adaxial side of the rosette leaves, whereas they are found on both adaxial and abaxial surfaces in the adult vegetative phase. During the reproductive stage, the trichome number gradually decreases on the main inflorescence stem and flowers (Telfer et al., 1997). Trichomes originate from the protodermal cells of the developing leaf primordia. The protodermal cells destined to become trichomes cease to divide and enter an endoreduplication cycle in which DNA replication continues in the absence of nuclear and cellular division. The mature trichome has a stalk with two to three branches and an average DNA content of 32C, suggesting that trichome cells undergo at least four rounds of endoreduplication during development (Hulskamp et al., 1994; Schnittger and Hulskamp, 2002). Over 30 different genes are known to control the developmental processes (Schellmann and Hulskamp, 2005). Extensive genetic and molecular analyses suggest that a network of transcription factors (TFs), belonging to three major groups: the R2R3 MYBs, the basic helix-loop-helix (bHLH) factors, and the WD40 repeat (WDR) protein, plays a crucial role in trichome development. These three groups of TFs form a trimeric activator complex, MYB-bHLH-WDR (MBW) that positively regulates the expression of downstream targets, which, in turn, induces trichome formation. The single repeat R3 MYBs act as negative regulators of trichome development. They compete with the R2R3 MYB to bind the bHLH factors and form a repressor complex (Serna and Martin, 2006; Ishida et al., 2008; Yang and Ye, 2013; Wang and Chen, 2014). Additionally, a group of C2H2 zinc finger TFs has been implicated in trichome formation on inflorescence stems and flowers in *Arabidopsis* (Gan et al., 2006, 2007a; Zhou et al., 2011, 2013).

Phytohormones, including gibberellins (GA), cytokinins(CK), and jasmonic acids (JA), are involved in numerous developmental processes in plants. These phytohormones also play a crucial role in trichome development in *Arabidopsis* (Chien and Sussex, 1996; Perazza et al., 1998; Dill and Sun, 2001; Traw and Bergelson, 2003; Gan et al., 2007a, b; Maes et al., 2008; Yoshida et al., 2009; Qi et al., 2011, 2014). In *Arabidopsis*, microRNAs have been shown to be involved in trichome development by controlling the expression of key regulatory genes (Yu et al., 2010). Recently, a posttranslational control mechanism has been implicated in trichome development (Patra et al., 2013a, b). Here, we focus on transcriptional regulatory network involved in the development of *Arabidopsis* trichomes. Furthermore, we discuss the influence of different phytohormones and their interactions on gene expression affecting trichome formation. The role of miRNA and 26S/ubiquitin proteasome system (UPS) in trichome development is also briefly discussed.

## TRANSCRIPTION FACTOR COMPLEX IN TRICHOME DEVELOPMENT

In *Arabidopsis*, trichome development is regulated by a transcriptional network involving several groups of TFs, namely, the MYB, bHLH, WDR, and C2H2 zinc finger proteins. The R2R3 MYB family in *Arabidopsis* is comprised of 126 members. Based on the conservation of the DNA binding MYB domain and the amino acid motifs in C-terminal domain, the R2R3 MYBs are divided into 25 sub-groups (Stracke et al., 2001; Dubos et al., 2010). The R2R3 MYBs belonging to sub-group 15, MYB0/GLABROUS1 (GL1) and AtMYB23/MYB23, are involved in trichome development (Oppenheimer et al., 1991; Kirik et al., 2001). These two proteins are functionally equivalent during trichome initiation but not during trichome branching (Kirik et al., 2005). Recently, a newly characterized R2R3MYB, AtMYB82, that does not belong to sub-group 15, has also been shown to regulate trichome development (Liang et al., 2014). Expression of *AtMYB82* under the control of the *GL1* promoter complements the trichome defect of the *gl1* mutant, suggesting that both GL1 and AtMYB2 are functionally equivalent.

The bHLH TF family is one of the largest known groups of TFs in *Arabidopsis* with more than 160 members divided into 12 subgroups (Heim et al., 2003). GLABROUS3 (GL3) and ENHANCER OF GLABROUS3 (EGL3), members of subgroup IIIf, are involved in trichome development in a partially redundant manner (Payne et al., 2000; Zhang et al., 2003). Mutation in the *GL3* locus results in fewer trichomes and reduced branching. The nuclei in *gl3-1* mutants undergo three, rather than four, rounds of endoreduplication, and this correlates with reduced trichome branching observed in this mutant. The *EGL3* locus has a moderate effect on trichome number. However, *gl3 egl3* double mutants have a glabrous phenotype. In addition to GL3 and EGL3, TRANSPARENT TESTA 8 (TT8) and AtMYC1, other members of the bHLH subgroup IIIf, have also been shown to affect trichome development (Maes et al., 2008; Symonds et al., 2011; Zhao et al., 2012). TT8 controls anthocyanin and proanthocyanidin (PA) biosynthesis in vegetative tissues and the seed coat (Nesi et al., 2000; Baudry et al., 2004). Maes et al. (2008) have demonstrated that TT8 also controls trichome development on leaf margins in *Arabidopsis*. *AtMYC1* mutants have less trichomes, compared with the wild type, indicating AtMYC1 acts as a positive regulator of trichome initiation (Zhao et al., 2012).

WD40 repeat proteins contain highly conserved 40–43 amino acid tandem repeats usually ending with Trp-Asp (WD). They are involved in the regulation of a number of processes, including cell cycle, cell fate determination, and cell signaling (Neer et al., 1994). In *Arabidopsis*, the WDR gene, *TRANSPARENT TESTA GLABRA 1* (*TTG1*), is a single copy gene (Walker et al., 1999). The *ttg1* mutant has pleiotropic phenotype, which is glabrous and deficient in anthocyanin accumulation (Walker et al., 1999).

In addition to the R2R3 MYBs, a group of seven R3 MYBs, that include TRIPTYCHON (TRY; Schnittger et al., 1999; Schellmann et al., 2002), CAPRICE (CPC; Wada et al., 1997), ENHANCER OF TRY, and CPC 1 (ETC1, ETC2 and ETC3; Kirik et al., 2004a, b; Wester et al., 2009), and TRICHOMELESS 1 (TCL1 and TCL2; Wang et al., 2007; Gan et al., 2011), are also involved in trichome development. Analyses of loss-of-function mutants reveal that these R3 MYBs act as negative regulators. Loss-of-function mutation in *CPC* causes increased trichome density (Schellmann et al., 2002) whereas mutation in *TRY* results in a trichome clustering phenotype (Schnittger et al., 1999; Schellmann et al., 2002). Mutation in *ETC1* does not dramatically affect the trichome phenotype whereas mutation in *ETC2* or *ETC3* results in increased trichome numbers. The higher order *ETC* mutants (*etc1 etc3* and *etc1 etc2 etc3*) exhibit significantly higher numbers of trichome compared to the respective single or double mutants, suggesting a redundant function by these regulators in trichome development (Kirik et al., 2004a, b; Wester et al., 2009). Loss-of-function mutations in the *TCL1* or *TCL2* locus result in ectopic trichome formation on inflorescence stems and pedicels (Wang et al., 2007; Gan et al., 2011). The number of trichomes on inflorescence stems and pedicels increase significantly in the *cpc tcl1* double mutant. The higher order *cpc etc1 etc3 tcl1* quadruple mutant, exhibits more trichomes on internodes and pedicels, compared to the *tcl1* or *cpc tcl1* double mutant, suggesting a role of CPC, ETC1 and ETC3 in trichome formation on inflorescence stems and pedicels (Wang et al., 2007, 2008).

The MYB (GL1)-bHLH (GL3/EGL3)-WDR (TTG1) proteins form a trimeric MBW complex that activates the expression of the homeodomain protein, GLABROUS2 (GL2; Rerie et al., 1994), which, in turn, induces trichome formation. GL3 contains three different protein-protein interaction domains: the N-terminal MYB-interacting region (amino acid 1-97) that interacts with GL1/CPC/TRY, the middle portion that includes the transactivation domain (amino acid 212-401) interacting with TTG1, and the C-terminal bHLH and ACT-like domain (amino acid 400-637) that homo/heterodimerize (Payne et al., 2000; Zhang et al., 2003). Recent studies demonstrate that the C-terminal domain of GL3/EGL3 also interacts with a number of factors involved in phytohormone signaling and protein degradation (Qi et al., 2011, 2014; Patra et al., 2013b). The MYB TFs contain a conserved amino acid signature motif, [D/E]Lx_2_[R/K]x_3_Lx_6_Lx_3_R, that is crucial for interaction with the bHLH proteins (Zimmermann et al., 2004). Physical interaction between GL1 and TTG1 has not been demonstrated. These findings suggest that the bHLH factors act as docking sites for a number of regulatory proteins, including the MYB and WDR proteins in the MBW complex. AtMYC1, like GL3/EGL3, has also been shown to interact with GL1 and TTG1, but does not dimerize. An arginine (Arg173) residue in AtMYC1 is found to be critical for its interaction with GL1. This Arg residue is conserved in GL3 and EGL3, and is essential for interaction with MYB partners (Zhao et al., 2012). The R3 MYBs typically lack the activation domain and compete with the R2R3 MYB, GL1, to bind GL3/EGL3, and form a repressor complex, thereby affecting the expression of downstream targets. There is a marked difference in the ability of these single repeat MYBs to disrupt the GL1-GL3 interaction. Using yeast three hybrid assay, CPC has been demonstrated as the most potent inhibitor of this activation complex, followed by ETC1, TRY, ETC2, and ETC3 (Wester et al., 2009). In a protoplast assay, TCL1 has been shown to be stronger than CPC in binding to GL3 (Wang et al., 2008). Using YFP-tagged CPC and ETC3, it has been demonstrated that these R3MYBs move from cell-to-cell and the strong binding of CPC to GL3 affects the mobility of CPC (Wester et al., 2009). Although the cell-to-cell movement signature motif (WxM) is conserved in all R3MYBs (Wang and Chen, 2014), the movement of TCL1 has yet to be experimentally demonstrated. Whether the strong interaction of TCL1 with GL3 affects its movement and biological activity still remains to be elucidated.

TRICHOMELESS 1 binds the *GL1* promoter in a chromatin immunoprecipitation (ChIP) assay and negatively regulates *GL1* expression (Wang et al., 2007). TCL1 probably acts as a negative regulator of trichome development by affecting the expression of *GL1*, as well as competing with GL1 for binding to GL3. Although the expression of most single repeat MYBs appears to be regulated by the MBW complex (Morohashi et al., 2007; Morohashi and Grotewold, 2009), it is unclear whether MBW also controls *TCL1* (Wang et al., 2007; Wang and Chen, 2014).

The C2H2 zinc finger proteins constitute one of the largest families of regulatory proteins, with 176 members in *Arabidopsis*, and are involved in numerous developmental and physiological processes in plants (Englbrecht et al., 2004). GLABROUS INFLORESCENCE STEMS (GIS), GIS2, ZINC FINGER PROTEIN 8 (ZFP8; Gan et al., 2006, 2007a), ZFP5 (Zhou et al., 2011, 2012), and ZFP6 (Zhou et al., 2013), which encode C2H2 zinc finger TFs, are involved in trichome formation in inflorescence stems and floral organs. Gene expression analyses of knockout mutants reveal a transcriptional hierarchy with ZFP6 acting upstream of ZFP5 which regulates the expression of *GIS*, *GIS2*, and *ZFP8*. GL1 and GL3, key TFs in the MBW complex, function further downstream of GIS2 and ZFP8 (Zhou et al., 2013).

Collectively, these findings suggest that a regulatory loop involving a group of activators and repressors fine tunes the expression of downstream gene targets and ultimately trichome formation. The activator complex (GL3/EGL3-GL1-TTG1) induces the expression of genes encoding the repressors (TRY/CPC) which can move into neighboring cells to form a repressor complex (GL3/EGL3-CPC/TRY-TTG1) and inhibit function of the activators. In addition to an activator–repressor mechanism, a depletion mechanism has also been proposed to operate, in parallel, during trichome development (Bouyer et al., 2008; Balkunde et al., 2011). In the depletion process, GL3 traps TTG1, resulting in depletion of TTG1 protein in neighboring cells. Consequently, cells with high levels of GL3 and TTG1 proteins are developed into trichomes. The depletion model is derived from the following findings: (a) TTG1 protein moves between cells, (b) TTG1 protein is preferentially accumulated in trichome initials and depleted in surrounding cells, and (c) depletion of TTG1 protein in neighboring cells, and its accumulation in trichome initials, is lost in the *gl3* mutant. Supporting this model, Balkunde et al. (2011) show that GL3 controls TTG1 movement, and interaction between GL3 and TTG1 is necessary for intracellular movement and epidermal distribution.

## PHYTOHORMONES AND TRICHOME DEVELOPMENT

Phytohormones, including GA, JA, and CK, play pivotal roles in controlling a wide array of biological processes in plants. Accumulating evidences suggest that these phytohormones are also crucial in trichome development. Here, we discuss the influence of different phytohormones and underlying molecular mechanisms, which control trichome formation in *Arabidopsis*.

Gibberellin is known to regulate a number of developmental processes in plants including seed germination, hypocotyl elongation, flowering, and trichome development. Evidence for the involvement of GA in trichome development comes from the analyses of several mutants in GA biosynthesis and signaling pathways in *Arabidopsis*. The GA biosynthesis mutant *ga1-3* has completely glabrous leaves, and application of exogenous GA to these plants restores trichome development. Additionally, GA stimulates the expression of *GL1*, and relative to wild type plants, the *ga1-3* mutant contains less *GL1* transcripts (Perazza et al., 1998). *SPINDLY* (*SPY*) is a repressor of GA signaling in *Arabidopsis*. Mutation in the *SPY* locus results in increased trichome formation (Chien and Sussex, 1996; Perazza et al., 1998). The *Arabidopsis* DELLA proteins are inhibitors of GA signaling and encoded by a family of five genes: *GIBBERELLIC ACID INSENSITIVE* (*GAI*), *REPRESSOR OF ga1-3* (*RGA*), and three *RGA-LIKE* genes (*RGL1*, *RGL2*, and *RGL3*). Among the five DELLA proteins, RGA, and GAI play significant roles in trichome formation. Mutations in *RGA* and *GAI* restore trichome initiation in the *ga1-3* mutant (Dill and Sun, 2001). Consistent with this, the expressions of several trichome regulators, including *GL1* and *GL3*, are up-regulated in *DELLA*-defective *ga1-3* mutants, whereas conditional over-expression of *RGA*-*GR* (RGA fused to rat glucocorticoid receptor) in these mutants reduces the expression of these trichome regulators (Gan et al., 2007b). Maes et al. (2008) have demonstrated that GA stimulates trichome formation through up-regulation of key TF genes. Expression of *GL1*, *MYB23*, *GL3* and *EGL3* are induced, whereas expression of *TRY*, *ETC1*, and *ETC2* are reduced, in response to GA treatment. *TTG1* expression is not significantly affected following GA treatment (Maes et al., 2008). Taken together, these findings suggest that GA regulates trichome formation by modulating the expression of key regulatory genes. These conclusions are further substantiated by the elegant demonstrations that individual TFs in the MBW complex are direct targets of DELLA proteins (Qi et al., 2014). RGA or RGL2 physically interact with GL3, EGL3, and GL1 to repress the transcriptional function of the MBW complex. Furthermore, analysis of the DELLA mutant, *penta* (*gai-t6 rga-t2 rgl1-1 rgl2-1* wild type for *RGL3*), *gl3 egl3* double mutant, and *penta gl3 egl3* mutants, suggest that the MBW complex acts downstream of DELLA proteins. Based on these findings, it is proposed that upon perception of the GA signal, protein–protein interactions between DELLA-GL1/GL3/EGL3 are disrupted as DELLA proteins are recruited to the SCF^SLY^ complex, and subsequently degraded by the 26S proteasome system. GL3, EGL3, and GL1 are consequently released to form a complex with TTG1 and activate the expression of GL2, which, in turn, mediates trichome formation.

Recent studies suggest that TFs other than MYB and bHLH proteins, also operate in the GA signaling pathway to regulate trichome development in inflorescence stems and flowers. The expression of C2H2 zinc finger TF genes, *GIS, GIS2, ZFP8, ZFP5, and ZFP6*, is stimulated in response to exogenous application of GA (Gan et al., 2006, 2007a, b; Zhou et al., 2013).

Jasmonic acid and its derivatives, collectively known as jasmonates (JAs), act as key signaling molecules that regulate numerous developmental processes in plants. JA biosynthesis is triggered in response to a variety of signals including wounding. Traw and Bergelson (2003) have shown that mechanical wounding and JA significantly induce trichome development in plants. The *Arabidopsis aos* mutant, deficient in JA biosynthesis due to a knock-out mutation in the *ALLENE OXIDE SYNTHASE* (*AOS*) gene, produces less trichomes compared to wild type plants and this defect can be rescued by JA treatment (Yoshida et al., 2009). Maes et al. (2008) have also demonstrated that JA stimulates trichome development by modulating the expression of several regulatory genes. In *Arabidopsis*, the F-box protein CORONATINE INSENTIVE1 (COI1), along with ASK1/ASK2, Cullin1, and Rbx1, form the SCF^COI^ complex that mediates JA signal transduction (Xie et al., 1998; Devoto et al., 2002). JA induction of trichome formation is attenuated in the *coi1-2* mutant, which is defective in JA signaling and produces less trichomes compared with wild type plants (Qi et al., 2011). Collectively, these findings show that JA plays an important role in trichome formation. Recent findings by Qi et al. (2011) elucidate the molecular mechanism underlying this process. They demonstrate that the MBW complex is involved in JA-induced trichome development in *Arabidopsis*. The expression of *GL3* and *GL1* is significantly induced in response to JA treatment in wild type plant, but severely weakened in the *coi1-2* mutant. Moreover, GL3 and GL1 physically interact with the JA-ZIM domain (JAZ) proteins in yeast, as well as plant cells. The JAZ proteins are known negative regulators of JA signaling. They are recruited to the SCF^COI^ complex upon perception of the JA signal and are subsequently degraded by the 26S proteasome system (Chini et al., 2007; Thines et al., 2007). These observations suggest that, in the absence of JA, the JAZ proteins bind to GL3, EGL3, and GL1 and inhibit the formation of the MBW complex. Upon perception of the JA signal, the JAZ proteins are degraded by the 26S proteasome system, thereby releasing GL3, EGL3, and GL1 to form the complex with TTG1, and activate the downstream targets to promote trichome formation.

Cytokinin (6-benzylaminopurine, BAP) acts as a positive regulator of trichome development in *Arabidopsis* (Maes et al., 2008). Plants treated with BAP produce more trichomes per leaf; however, the trichomes are shorter and nuclear DNA content is less than in untreated plants, indicating that BAP affects endoreduplication in trichomes. The expression of *GL1*, *MYB23*, *GL3*, and *EGL3*, is also stimulated following BAP treatment. The expression of *GIS2*, *ZFP5*, *ZFP8*, and *ZFP6*, that regulate trichome formation on inflorescence stems, is also influenced by cytokinins (Gan et al., 2007a; Zhou et al., 2013).

Trichome formation is also affected by brassinosteroids (BR), ethylene (ET), and salicylic acid (SA). The *Arabidopsis bls1* mutant, impaired in BR response, develops fewer trichomes on both abaxial and adaxial surfaces of the leaf, indicating a possible involvement of BR in trichome development (Laxmi et al., 2004). The ET receptor mutant *etr2-3*, has completely unbranched trichomes. Through epistatic and gene expression analysis, it has been shown that the ET signaling cascade involves CHROMATIN ASSEMBLY FACTOR 1 (CAF1) and TRY to control trichome branching, and is independent of the GL3, GL2 pathway (Plett et al., 2009). Application of SA significantly reduced the number and density of trichomes in different cultivars of *Arabidopsis*, indicating its negative regulatory role in trichome development (Traw and Bergelson, 2003).

## PHYTOHOMONE CROSS-TALK AND TRICHOME DEVELOPMENT

Phytohormones are known to act synergistically as well as antagonistically to regulate different developmental processes in plants. The antagonistic action of GA and JA regulate hypocotyl elongation, root growth, and flowering (Hou et al., 2010, 2013; Yang et al., 2012), whereas their synergistic action regulates stamen development and trichome formation (Traw and Bergelson, 2003; Song et al., 2013; Qi et al., 2014). Trichome density and number are significantly higher in plants treated with a combination of GA and JA compared with plants treated with only JA. Consistent with this, the expression of *GL2* and *MYB23* are found to be significantly up-regulated by combined treatment of GA and JA. Moreover, the GA biosynthetic inhibitor, paclobutrazol, represses JA-induced trichome formation and expression of *GL2* and *MYB23*. JA-induced trichome formation is also attenuated in the GA biosynthesis mutant *ga1-3*. Similarly, the *coi1-1* mutant, defective in JA signaling, inhibits GA-induced trichome formation. Recent studies by Qi et al. (2014) reveal the molecular mechanism underlying the GA-JA synergy in trichome development. Both JAZ and RGA (DELLA protein) bind to trichome regulators, GL3, EGL3, and GL1. GA and/or JA signals control the level of these repressor proteins via 26S proteasome-dependent proteolysis and maintain the stable transcription of the activators that induce trichome formation.

The positive regulatory role of GA and CK on trichome development is well documented. A recently identified C2H2 zinc finger TF, ZFP6, seems to function as an integrative hub of GA and CK signals in promoting trichome formation in *Arabidopsis*. *ZFP6* expression is induced in response to GA and CK treatment. Moreover, GA- and CK-induced expression of the downstream targets of ZFP6, *ZFP5*, *ZFP8*, and *GL1*, is significantly affected in *zfp6* mutant (Zhou et al., 2013).

The negative cross-talk between JA and SA-dependent pathways in *Arabidopsis* is well documented. These phytohormones act antagonistically to regulate trichome development. *Arabidopsis* plants treated with a combination of JA and SA produce lower numbers of trichomes compared with plants treated with JA alone (Traw and Bergelson, 2003).

## microRNA AND TRICHOME DEVELOPMENT

microRNAs (miRNAs) are small endogenous non-coding RNAs of 20–22 nt in length and present in plants, animals, and protozoa. miRNAs modulate the expression of their target genes at the posttranscriptional level and thus control many aspects of cellular functions (Voinnet, 2009; Fabian et al., 2010). Recent studies indicate that miRNAs regulate trichome development by modulating the expression of *SQUAMOSA PROMOTER BINDING PROTEIN LIKE* (*SPL*) genes (Yu et al., 2010). SPLs are a group of plant-specific TFs that share a highly conserved SBP DNA-binding domain first identified in a protein that binds to the promoter of the *SQUAMOSA* gene of *Antirrhinum majus* (Cardon et al., 1999). SPLs regulate numerous fundamental aspects of plant growth and development, including phase transition, trichome distribution and flowering (Chen et al., 2010). In *Arabidopsis*, SPLs are negative regulators of trichome development in inflorescence stem and floral organs. The *SPL* gene family has 17 members, 10 of which are targeted by miRNA156 (Rhoades et al., 2002). Expression of miRNA156 and *SPLs* (*SPL3*/*SPL9*) are temporally regulated (Wu and Poethig, 2006; Yu et al., 2010). miRNA156 levels are highest in young plants and decline as the plant ages. On the contrary, expression of *SPLs*, targets of miRNA156, is low in young plants and increase gradually during the reproductive stage. The expression pattern fits well with the gradual loss of trichomes on stem and floral organs. Consistent with this, plants expressing a mimicry target of miR156 accumulate significant *SPL* transcripts and show a reduction in trichome density on stems. Similar results are found when miR156-resistant forms of *SPL3*, *SPL10*, *SPL13*, and *SPL9* are over-expressed in plants. By comparison, transgenic *Arabidopsis* plants constitutively expressing miRNA156 produce ectopic trichomes on stem and floral organs. Moreover, SPL9 directly binds to the promoters of *TCL1* and *TRY*, negative regulators of trichome development, and activates their expression in a GL1-independent manner, leading to reduced trichome formation (Yu et al., 2010). Together, these observations suggest that the temporal control of trichome development in *Arabidopsis* is regulated by the miR156-targeted SPL TFs.

## UBIQUITIN/26S PROTEASOME SYSTEM AND TRICHOME DEVELOPMENT

The 26S proteasome is a multi-subunit ATP-dependent protease complex assembled from two particles: the 20S core particle (CP) and the 19S regulatory particle (RP; Zwickl et al., 1999). The UPS-dependent proteolysis is the most elaborate and complex regulatory mechanism controlling activities of short-lived proteins in eukaryotes. Over 1300 genes in the *Arabidopsis* genome are associated with the 26S proteasome pathway (Vierstra, 2003). Loss-of-function mutations in one of the RP components, RPT2a, result in several physiological abnormalities including aberrant trichome development. The *rpt2a* mutant has larger trichomes with increased branch number. Additionally, trichomes of *rpt2a* plants have larger nuclei compared with the wild type, suggesting RPT2a is involved in regulation of endoreduplication in trichomes (Sonoda et al., 2009).

26S/ubiquitin proteasome system comprises E1, E2, and E3 enzymes that act coordinately to conjugate ubiquitin moieties to the target proteins and pave the way for subsequent degradation by the 26S proteasome. E3 enzymes determine substrate specificity by recognizing a single or small group of proteins and in plants, are divided into two subgroups, RING/U boxes and HECT ubiquitin ligases (Smalle and Vierstra, 2004). In *Arabidopsis*, mutation in the UPL3/KAK locus, which encodes a HECT domain E3 ligase, results in trichomes with increased branch number and higher nuclear DNA content, suggesting that UPL3 regulates of ploidy level in trichomes by controlling the activities of proteins that normally promote the endoreduplication cycle (Downes et al., 2003; El Refy et al., 2003). GL3 is a potential target of UPL3 because it is a positive regulator of endoreduplication in trichomes, and the supernumerary trichomes in *upl3*/*kak* seedlings is suppressed in *kak gl3* double mutant background (Sako et al., 2010). Our recent demonstration, that GL3 and EGL3 are short-lived proteins, supports the hypothesis that GL3/EGL3 are targets of UPL3. UPL3 physically interacts with GL3 and EGL3, and mediates degradation via UPS (Patra et al., 2013b). Additionally, we have also shown that TTG1 and TT8, the regulator of trichome formation on leaf margin, are targets of UPS. However, the specific E3 ligase that mediates this degradation remains to be identified (Patra et al., 2013a). Endoreduplication cycles in *Arabidopsis* trichomes are also controlled by another class of ubiquitin ligases, the RBX1-containing Cullin-RING E3 ubiquitin ligases (CRLs). Of five known cullin genes, *CUL1*, *CUL3A*, and *CUL4* are strongly expressed in young trichomes. *CUL1* and *CUL3A* loss-of-function mutants are phenotypically indistinguishable from wild type. However, a knock-down line of *CUL4* produces small trichomes with less nuclear DNA content. The CUL4-CRL complex modulates cyclin-dependent kinase (CDK) activity presumably by mediating the degradation of a class of CDK inhibitors during endoreduplication cycles (Roodbarkelari et al., 2010).

## CONCLUDING REMARKS

During the past decade, substantial and significant progress has been made in delineating the elaborate gene regulatory network that controls trichome development in *Arabidopsis*. Multiple lines of evidence suggest that a number of transcriptional activators and repressors act in concert to fine tune the spatial and temporal distribution of trichomes. Additionally, phytohormones such as JA, GA, and CK, act synergistically or antagonistically to modulate the expression of genes encoding these regulators. Recent findings, pertaining to miRNAs and 26S/UPS-dependent regulation of TFs in trichome development, highlight the complexity of the regulatory network. Whether other regulators such as GL1/MYB23 and R3 MYBs are targets of miRNA and/or 26S/UPS remains to be elucidated.

Our current knowledge about the gene regulatory network is largely limited to the unicellular non-glandular trichomes in the model plant, *Arabidopsis*. Very little is known about the regulatory network that controls the development of glandular secretory trichomes. This type of trichomes is found in many plants, including tobacco, tomato, basil, and mint, and is thus an important source of phytochemicals. Over-expression of *GL1* in tobacco does not augment trichome formation, suggesting that different regulatory mechanisms control trichome development in *Arabidopsis* and tobacco (Payne et al., 1999). Recently, an integrated genomic database, TrichOME (www.planttrichome.org), has been developed. The database hosts more than a million EST sequences from both trichome and corresponding non-trichome tissues from 13 species, including tobacco, basil, and mint, and provides a potential source for genes involved in development of glandular and non-glandular trichomes (Dai et al., 2010). Understanding the regulatory network that controls the development of multicellular trichomes will aid our efforts to engineer trichomes to produce commercially important phytochemicals.

## Conflict of Interest Statement

The authors declare that the research was conducted in the absence of any commercial or financial relationships that could be construed as a potential conflict of interest.
